# Development and Validation of an Analytical Method for Carnosol, Carnosic Acid and Rosmarinic Acid in Food Matrices and Evaluation of the Antioxidant Activity of Rosemary Extract as a Food Additive

**DOI:** 10.3390/antiox8030076

**Published:** 2019-03-26

**Authors:** Seung-Hyun Choi, Gill-Woong Jang, Sun-Il Choi, Tae-Dong Jung, Bong-Yeon Cho, Wan-Sup Sim, Xionggao Han, Jin-Sol Lee, Do-Yeon Kim, Dan-Bi Kim, Ok-Hwan Lee

**Affiliations:** 1 Department of Food Science and Biotechnology, Kangwon National University, Chuncheon 24341, Korea; zzaoszz@naver.com (S.-H.C.); jkw5235@naver.com (G.-W.J.); docgotack89@hanmail.net (S.-I.C.); lgtjtd@naver.com (T.-D.J.); bongyeon.cho92@gmail.com (B.-Y.C.); simws9197@naver.com (W.-S.S.); xionggao414@hotmail.com (X.H.); amylbl32@naver.com (J.-S.L.); doy0918@naver.com (D.-Y.K.); 2 Korea Food Research Institute, Wanju-gun 55365, Korea; dbkim1022@kfri.re.kr

**Keywords:** rosemary extracts, method validation, antioxidant activity, HPLC, food matrices

## Abstract

Antioxidants are used to prevent the oxidation of foods. When used for food additive purposes, the dosage should be regulated and the functionality evaluated to ensure stability. In this study, we performed a method validation for the quantitative analysis of rosemary extract residues and evaluated the antioxidant activity of rosemary extract in food matrices. The validated method was able to determine rosemary extract under the optimized high-performance liquid chromatography-photodiode array (HPLC-PDA) conditions. Furthermore, the antioxidant activity was evaluated by peroxide value, acid value, and in terms of the residual antioxidant levels in lard oil. For HPLC-PDA analysis, the limit of detection and quantification of rosemary extracts was ranged from 0.22 to 1.73 μg/mL, 0.66 to 5.23 μg/mL and the recoveries of the rosemary extracts ranged from 70.6 to 114.0%, with relative standard deviations of between 0.2% and 3.8%. In terms of antioxidant activity, carnosic acid performed better than carnosol. Furthermore, by evaluation of the residual antioxidant level using HPLC, we found that carnosic acid is more stable in lard oil than carnosol. These results indicate that rosemary extract can be used as an antioxidant and that the analytical method is suitable for the determination of rosemary extract in various food samples.

## 1. Introduction

Lipid oxidation is a major cause of quality degradation in fat-containing foods. All foods, especially those with high fats, are vulnerable to lipid peroxidation and free radical attack, leading to food decay and rotten odors. The formation of free radicals in food causes undesirable changes, destroying vitamins and other substances, reducing the nutritional value, and affecting the health, safety, color, flavor, and texture of the food [1,2]. Autoxidation of lipids generally involves a free radical chain reaction, which is initiated by exposure of the lipids to heat, ionizing radiation, light, metalloprotein catalysts, or metal ions. Thus, the inhibition of free radicals is of practical importance in protecting lipids from oxidation [3].

Antioxidants are widely used in foods to delay or prevent oxidation. Being easily oxidized, they exchange electrons with peroxides and free radicals, preventing these compounds from undergoing reactions with other substances in the food matrix that would lead to damage. The addition of antioxidants to foods prevents the formation of various flavors and rotten odors, thereby extending the shelf life [4,5,6].

*Rosmarinus officinalis L.* (Lamiaceae), commonly referred to as rosemary, is a plant used worldwide in cooking as an herb. Rosemary contains a large number of phenolinc compounds including phenolic diterpenes such as carnosol, carnosic acid, epi- and iso-rosemanols, rosmanol, and the phenolic ester, rosmarinic acid [7,8]. Rosemary extract also contains a large number of phenolic compounds, including carnosol, carnosic acid and rosmarinic acid, and has been widely used in the food industry due to its inherent high antioxidant activity [9]. In some countries, rosemary extract is designated as an antioxidant food additive, with defined acceptance criteria.

It is designated as “Rosemary Extract” (China) or “Extracts of rosemary” (European Union) in countries where it has been approved as a food additive. In China, the addition of rosemary extract is permitted to a maximum concentration of ~300.0–700.0 μg/mL, depending on food type. In the European Union, the maximum residue level of rosemary extract is defined by the sum of carnosol and carnosic acid, since these are the main active ingredients of rosemary extract: Depending on the type of food, ~30.0–250.0 μg/mL is permitted [10,11]. However, in South Korea, the addition of rosemary extract to food is not permitted. Consequently, there is a need for a validated analytical method for detecting the addition of rosemary extract as an unauthorized antioxidant, in foods commonly consumed in South Korea. There have been no studies on the analysis of rosemary extract to evaluate its content as a food additive in permitted food types. Previous studies on the analysis of rosemary have been reported, such as the analysis of active ingredients including carnosol and carnosic acid in extracts, and the analysis of active ingredients in rosemary [8,12]. If rosemary extract is used as a food additive, to ensure food safety its concentration should be monitored to determine whether it exceeds the allowable level. Therefore, an analysis method that is capable of accurately analyzing rosemary extract in foods is required. However, to date, no research has been conducted into the development of an analytical method to determine rosemary extract in food matrices. 

The addition of an antioxidant to food prevents the food from oxidizing during storage; over time, the antioxidant loses its activity and its concentration decreases. The residual antioxidant level is one of the important factors in the role of antioxidants in food. However, most previous studies have evaluated the antioxidative activity of the major active substances of rosemary extract, and, to date, no studies have investigated the antioxidant activity with residual levels of rosemary extract in food [13,14].

In this study, we developed and validated a high-performance liquid chromatography-photodiode array (HPLC-PDA) method for the determination of rosemary extract in various primary domestic and imported food product matrices, including edible oils, processed meat products, and dressings. Also, to demonstrate the effective application of the established method on real samples, various edible oil, processed meat product, and dressing samples were collected from grocery markets in South Korea and their rosemary extract contents were determined. In addition, to evaluate its function as an antioxidant, rosemary extract was added to lard oil and the storage stability was evaluated by measuring the residual antioxidant level, peroxide value and acid value.

## 2. Materials and Methods

### 2.1. Materials

Analytical standards, such as rosmarinic acid, carnosol, and carnosic acid, were obtained from Sigma-Aldrich (St. Louis, MO, USA). All solvents were suitable for analysis and were purchased from J.T. Baker (Phillipsburg, NJ, USA).

### 2.2. Food Materials

Ninety food samples including thirty edible oils, thirty processed meat products, and thirty dressings, were purchased in local markets in South Korea. The shelf life of the samples was sufficient for the investigation. To validate the procedure, edible oils, processed meat products and dressings that were found to be free of rosemary extract, were selected. Prior to analysis, all samples were stored under the storage conditions indicated on the product label.

### 2.3. Optimization of HPLC Conditions

Rosemary extracts were tested using HPLC, and the optimum analytical conditions were determined. We first evaluated the comparison of the different analytical methods, as it achieved the best results. Then, analytical parameters such as column type and column temperature were evaluated to provide optimum separation conditions for rosemary extract in edible oils, processed meat products and dressings. The sensitivity of the analytical method was evaluated based on the maximum allowable levels of rosemary extract in foreign countries (within the European Union and China).

### 2.4. Optimization of Extraction Method

Using the optimized HPLC-PDA analysis method, the optimum preparation method for removing rosemary extract from three matrices (edible oil, processed meat products, and dressing) was determined. The recovery was used as an index for establishing the optimal sample preparation method. Sample preparation for rosemary extract analysis was optimized using previously reported methods [15,16].

### 2.5. Sample Preparation

The samples were prepared according to the modified method of Kim et al. (2016) [15]. The samples were weighed (5 g each) into a 50 mL conical tubes, rinsed with 15 mL of n-hexane and then transferred to a separatory funnel containing 5 mL of n-hexane, followed by extraction with 150 mL portions of n-hexane-saturated acetonitrile. For preparing the processed meat products and the dressing samples, the organic solvent layer was transferred to another separatory funnel to remove the residues which interfered with the separations. The acetonitrile phase was collected in a concentrate flask, and then evaporated to a volume of 3 to 4 mL using a water bath (≤40 °C, EYELA, SB-1200, Tokyo, Japan) with a vacuum rotary evaporator (EYELA, N-1200A, Tokyo, Japan). The flask was rinsed with small portions of solvent (acetonitrile:iso-propanol, 1:1, *v/v*) which were then transferred to a 10 mL volumetric flask. The rinsing step was repeated until exactly 10 mL was collected in volumetric flask. The samples were filtered through a 0.45 μm syringe filter (Millex-HV, Millipore, Bedford, MA, USA).

### 2.6. HPLC Instrument Conditions

The HPLC apparatus was a Waters 2695 separation module HPLC system (Waters Co., Milford, MA, USA) equipped with a pump, an autosampler, a column oven, and a Waters 996 photodiode array detector. The analytical column was a Shiseido Capcell Pak C_18_ UG120 (Shiseido, 4.6 mm × 250 mm, 5.0 μm, Tokyo, Japan). The column temperature was maintained at 30 °C. The mobile phase was composed of A (1% acetic acid in water) and B (methanol) with a gradient elution as follows: 0–20 min, linear from 10 to 65% A; 20–40 min, linear from 65 to 100% A; 40–45 min, maintained at 100% A; 45–47 min, linear from 100 to 10% A; and then finally, holding for 3 min. The mobile phase was filtered through a 0.45 μm membrane filter (Whatman, Amersham, UK) and degassed under vacuum. The flow rate was set 1.0 mL/min, and the injection volume was 20 μL. The antioxidants were determined at 284 nm. Data acquisition and remote control of the HPLC system were performed using Empower software (Waters Co., Milford, MA, USA).

### 2.7. Method Validation

The HPLC method for the determination of rosemary extract in three food matrices (edible oils, processed meat products, and dressings) was validated in terms of linearity, trueness, precision, limit of detection (LOD) and quantification (LOQ), according to the guidelines of the International Conference on Harmonization (2005) [17]. The matrix-matched calibration curves were prepared by spiking prepared extracts of blank edible oil, processed meat product, and dressing in seven concentrations from 1.56 to 100.0 μg/mL for rosmarinic acid, and from 6.25 to 400.0 μg/mL for carnosic acid and carnosol. During analytical method development and validation, matrix-matched calibration curves from each assessed analytical method were evaluated to ensure that the sensitivity and linearity were consistent with the experimental observations, and that there were no matrix peak interferences. The linearity evaluation was performed by calculating the linearity of the calibration curve determined using a range of concentrations of each compound. The selectivity was assessed by examining the chromatogram to confirm that there were no ingredients that could interfere with the target analyte of rosemary extract in edible oils, processed meat products, and dressings. The LOD and LOQ were calculated for the analytes in edible oils, processed meat products, and dressings. The trueness and precision were determined in three food matrices at three different concentration levels (rosmarinic acid: 5.0, 10.0, and 20.0 μg/mL; carnosic acid and carnosol 25.0, 50.0, and 100.0 μg/mL). Both inter-day (three repeats on three different days) and intra-day (three repeats during a single day) method validation experiments were performed.

### 2.8. Function Evaluation as an Antioxidant

#### 2.8.1. Experimental Condition

Lard oil was used to evaluate the functionality of the rosemary extract as an antioxidant. In other countries, rosemary extracts defined the sum of carnosol and carnosic acid, is permitted at 50 μg/mL in lard oil. Therefore, these two compounds, except rosmarinic acid, were used for the functional evaluation. Two antioxidant components of rosemary extract (carnosic acid and carnosol) were added to lard oil to provide a combined concentration of 50 μg/mL. In addition, to compare the effects of synthetic antioxidants (carnosol and carnosic acid standards) and natural antioxidants, rosemary leaf extract was prepared and analyzed by HPLC to calculate its carnosol and carnosic acid concentrations. This leaf extract was then added to lard oil to provide a combined carnosol and carnosic acid concentration of 50 μg/mL. The mixture of carnosol and carnosic acid standards was also added to lard oil to provide a combined concentration of 50 μg/mL. To provide a positive control, butylated hydroxyanisole (BHA) was added to lard oil at a concentration of 50 μg/mL. The prepared samples were stored in a dry oven at 50 °C for 42 days. The peroxide value, acid value, and residual antioxidant level of each sample were evaluated every 7 days.

#### 2.8.2. Measurement of Antioxidant Activity

Antioxidant activity was evaluated using the peroxide value and acid value. The peroxide value and acid value of each sample were analyzed by modifying the Official Methods and Recommended Practices of the American Oil Chemists’ Society (AOCS, 1993) Cd 8-53 and Te 1a-64, respectively [18]. The peroxide value was proceeded as follows. First, 2 g of sample was dissolved in 10 mL of acetic acid and chloroform mixture (3:2, *v/v*) in a 100 mL Erlenmeyer flask and 0.4 mL of KI saturated solution was added and kept in a dark place for 10 minutes. After that, 12 mL of distilled water and 0.4 mL of 1% starch solution were added in succession, and the mixture was shaken. The resultant was titrated with 0.01 N Na_2_S_2_O_3_ solution. Acid value was measured by the following method. 5 g of sample is precisely weighed, placed in a stoppered Erlenmeyer flask, and dissolved in 100 mL of a neutral ethanol/ether mixture (1:2). Using phenolphthalein solution as an indicator, titrate with 0.1 N potassium hydroxide ethanolic standard solution until pale red color persists for 30 s. The peroxide value and the acid value were measured every seven days. The peroxide value and the acid value results were respectively expressed as milli-equivalents of hydroperoxides per kg of lipids (meq/kg) and in terms of the number of mg of KOH required to neutralize the free fatty acids contained in 1 g of lipid (mg KOH/g).

#### 2.8.3. Measurement of Residual Antioxidant Level

The residual amount of antioxidant compound was evaluated using HPLC. Sample preparation was carried out as described in Section 2.5. The residual amount of antioxidant in each experimental group was expressed as the rate of change of antioxidant over time. The results of each test group measured on the first day are displayed as 100%, while the later results are displayed as a percentage of the first day’s result.

### 2.9. Statistical Analysis

The peroxide value, acid value, and residual antioxidant level results were analyzed by ANOVA and Duncan’s multiple range tests using SAS 9.4 (SAS Institute Inc., Cary, NC, USA). Significance was indicated at a *p*-value of < 0.05.

## 3. Results and Discussion

### 3.1. Optimization of HPLC Conditions

The chromatographic conditions for analyzing the rosemary extract were investigated and optimized. The sensitivity of the analytical method was determined based on the residual concentration of rosemary extracts permitted in foreign countries (China and within the European Union) [10,11]. The analytical method for the target analyte separation was based on gradient elution with acetonitrile, methanol or acetic acid solution as the mobile phase [19,20,21].

In the gradient elution method, using acetonitrile and acetic acid solution, ghost peak detection and tailing of the carnosic acid peak were problematic. The gradient elution method using methanol, acetonitrile, and acetic acid solution resulted in tailing of the carnosol and carnosic acid peaks at high concentration (data not shown). The gradient elution method using methanol and acetic acid solution provided better peak separation and peak shapes than those of the other methods. Accordingly, we selected the latter gradient elution method for determination of rosemary extract in food matrices. 

To compare the separation efficiency of the C_18_ columns from a range of manufacturers, we analyzed the standard solution (at low, medium, high concentration; rosmarinic acid: 5.0, 10.0, and 20.0 μg/mL; carnosic acid and carnosol 25.0, 50.0, and 100.0 μg/mL) and the sample blank. The column types used in this comparison were Shiseido Capcell Pak C_18_ UG 120 (4.6 × 250 mm, 5.0 μm), and Waters Sunfire C_18_ (4.6 × 250 mm, 5.0 μm). The retention times varied depending on the manufacturer of the C18 column. The Shiseido Capcell Pak C_18_ UG 120 showed better separation and the retention times of the analytes were faster than found with other C_18_ columns (data not shown). Therefore, we selected the Shiseido Capcell Pak C_18_ UG 120 column for further study.

The column temperature was adjusted to the separation efficiency of the analytes and the pressure inside the analytical device. Since the column temperature is an important factor that affects chromatographic separation, changes in column temperature can be useful for efficient separation [22,23,24]. As the temperature increases, the retention time of the peak decreases, without affecting component separation. Nevertheless, a column temperature of 30 °C was selected since the highest overall peak area was observed at this temperature (data not shown).

The analytical method for rosemary extract determination was optimized by comparing the results with different mobile phases, column manufacturers, and column temperatures. Finally, the optimal HPLC analytical conditions of rosemary extracts was described in Section 2.6.

### 3.2. Optimization of Extraction Method

The sample preparation is important in chromatographic applications. One of the main goals of this step is to concentrate the analyte to remove the interfering matrix components and particulates; therefore, increasing the sensitivity [25]. First, we assessed whether the method of Suh et al. (2011) could be applied to rosemary extracts [16]. This method reports the pretreatment of erythorbic acid, an antioxidant, in processed meat products. The antioxidant compound was extracted by liquid-liquid extraction using 2% metaphosphoric acid solution. The recovery rate results from this method showed that rosmarinic acid had a high recovery rate, but that carnosol and carnosic acid were not detected. 

**Figure 1:**
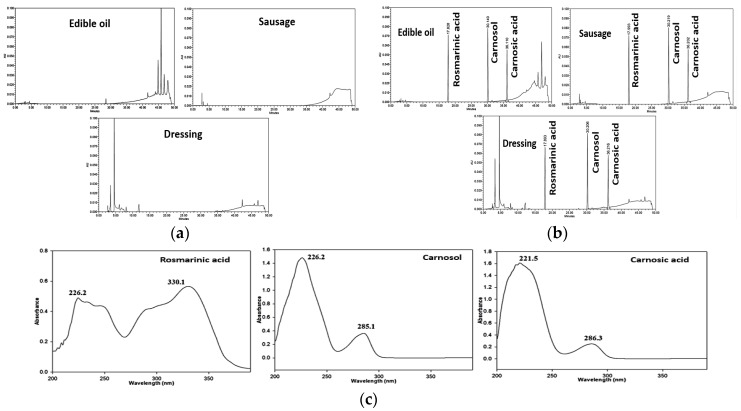
Chromatograms of rosemary extracts obtained using HPLC-PDA. (**a**) Blank samples; (**b**) Spiked samples; (**c**) Analyte spectra.

We also assessed the Kim et al. (2016) method, which analyzed seven synthetic antioxidants in edible oils [15]. The synthetic antioxidants were extracted by liquid-liquid extraction method using n-hexane-saturated acetonitrile as the extraction solvent. With this method, the recoveries of rosmarinic acid, carnosol, and carnosic acid were all excellent, and there was minimal use of organic solvents. However, when applied to the processed meat products and dressings, precipitates formed in the separating funnel; therefore, it was difficult to separate the organic layers efficiently. Consequently, we modified the pretreatment process to establish the optimal pretreatment conditions. For samples of the three food matrices, the rosemary extract recoveries ranged from 73.1 to 116.5% with the modified method. Therefore, we selected a sample preparation method modified from Kim et al. (2016), since this provided the highest rosemary extract recovery from the three matrices (Figure 1). The modified method is presented in Section 2.5.

### 3.3. Method Validation

#### 3.3.1. Selectivity, Linearity, LOD, and LOQ

Selectivity in chromatography refers to the extent to which an analytical method can determine the analyte in a matrix without interference from other components that are expected to be present in the food matrix [26]. The chromatograms of rosemary extract standards obtained using the HPLC-PDA method are shown in Figure 1. No interferences or co-eluting peaks are not observed and the analytes are separated efficiently in the chromatograms in edible oils, processed meat products, and dressings using the Capcell Pak C_18_ column. The linearity of the established method was evaluated in the range 1.56–100 μg/mL for rosmarinic acid, and 6.25–400 μg/mL for carnosol and carnosic acid. The correlation coefficients (R^2^) of the rosemary extracts were in the range of 0.9987–1.0000. For HPLC-PDA, the LOD and LOQ are shown in Table 1. The LOD lay in the range of 0.38–0.78 μg/mL in edible oils, 0.38–1.50 μg/mL in processed meat products, and 0.22–1.73 μg/mL in dressings. The LOQ lay in the range of 1.14–2.38 μg/mL in edible oils, 1.15–4.55 μg/mL in processed meat products, and 0.66–5.23 μg/mL in dressings. The sensitivity of the HPLC-PDA method is suitable for the quantitative analysis of rosemary extracts below the maximum acceptable levels in foreign countries (China and within the European Union).

#### 3.3.2. Trueness and Precision

The recovery results for the rosemary extracts are presented in Table 2. The trueness of the developed analytical method was determined from the recovery rates. The spiking levels for the recovery study were 5.0, 10.0, and 20.0 μg/mL for rosmarinic acid, and 25.0, 50.0, and 100.0 μg/mL for carnosol and carnosic acid in the edible oil, processed meat product, and dressing matrices. The average recoveries in edible oil were 70.6–90.8% for rosmarinic acid, 82.6–93.8% for carnosol, and 85.1–92.5% for carnosic acid. In processed meat products, the average recoveries were 72.8–106.9% for rosmarinic acid, 99.1–108.2% for carnosol, and 99.4–115.2% for carnosic acid. In dressings, the average recoveries were 80.4–116.3% for rosmarinic acid, 101.5–114.0% for carnosol, and 99.2–116.5% for carnosic acid. In Codex Alimentarius Commission Guidelines (CAC/GL 40), the average recovery criteria applicable to concentrations of residues at the levels ≥ 10 μg/mL ≤ 100 μg/mL and > 1 μg/mL ≤ 10 μg/mL are 70–120% and 60–120%, respectively [27]. The results of recovery satisfied this guideline.

The precision of the rosemary extract analysis was evaluated using the intra-day and inter-day RSDs. For all samples, the intra-day and inter-day precisions ranged respectively as follows: From 0.2 to 1.8% and from 0.2 to 3.6% for rosmarinic acid; from 0.2 to 0.8% and from 0.3 to 3.8% for carnosol; and, from 0.2 to 1.4% and from 0.8 to 3.1% for carnosic acid. The relative standard deviation (RSD) analysis results are in accordance with the CAC/GL 40. 

### 3.4. Sample Collection and Monitoring of Residual Rosemary Extract Levels

The method developed and validated in this study was used to monitor the presence of residual rosemary extracts in edible oils, processed meat products, and dressings in domestic markets. A total of ninety samples of edible oils, processed meat products, and dressings were collected from grocery markets in South Korea. Using the HPLC-PDA analysis method, chromatographic peaks in each sample were identified by comparison of the retention time and the PDA spectrum of the rosemary extract standard. Across the ninety analyzed samples, no rosemary extracts were found in any of the samples.

### 3.5. Assessment of Functionality as Antioxidant

#### 3.5.1. Antioxidant Activity

To evaluate the antioxidative activity of rosemary extract as a food additive, carnosol and carnosic acid were added to lard oil and the peroxide and acid values were evaluated. 

**Figure 2:**
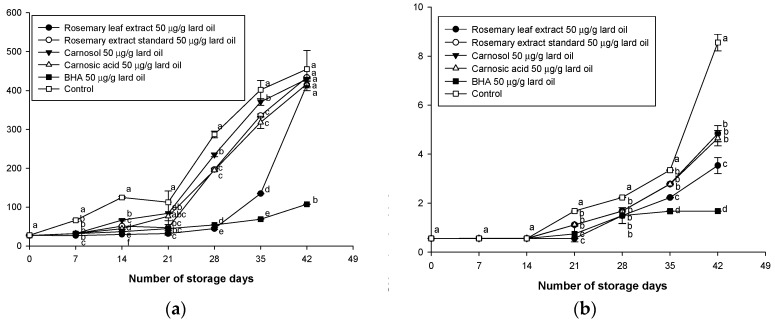
Antioxidant activity of rosemary extracts in lard oil during storage. (**a**) Peroxide value; (**b**) Acid value. ^a–d^ Different letters indicate statistically significant different among the groups at *p*-value < 0.05.

The antioxidant activity results for the rosemary extracts are shown in Figure 2. The peroxide value of the lard oil increased across the storage period, from 27.71 to 454.95 meq/kg in the control group (with no antioxidant added to the lard oil). The peroxide value of the lard oil with added antioxidants increased as follows: From 26.61 to 433.77 meq/kg in the carnosic acid + carnosol group; from 27.22 to 429.34 meq/kg in the carnosol group; from 26.84 to 416.71 meq/kg in the carnosic acid group; from 27.09 to 414.69 meq/kg in the rosemary leaf extract group; and, from 27.12 to 107.52 meq/kg in the BHA group. The acid value of the lard oil increased across the storage period, from 0.56 to 8.56 mg KOH/g in the control group (with no antioxidant added to the lard oil). The acid value of the lard oil with added antioxidants increased as follows: From 0.56 to 4.83 mg KOH/g in the carnosic acid + carnosol group; from 0.56 to 4.84 mg KOH/g in the carnosol group; from 0.56 to 4.65 mg KOH/g in the carnosic acid group; from 0.56 to 3.53 mg KOH/g in the rosemary leaf extract group; and, from 0.56 to 1.67 mg KOH/g in the BHA group. Acid value analysis showed that there were no significant differences between the experimental groups until day 14. However, a significant difference was observed between the experimental groups after day 21. The acid value was highest in the control group, followed by carnosol + carnosic acid group, carnosol group, carnosic acid group, rosemary leaf extract group, and butylated hydroxyanisole (BHA) group, showing a similar tendency to that observed for the peroxide value. In terms of the peroxide value and the acid value, between the two components of rosemary extracts used as acceptance criteria, carnosic acid was found to inhibit oxidation more effectively than carnosol. These results are in good agreement with reports by Frankle et al. (1996) and Hopia et al. (1996) who demonstrated that carnosic acid has superior antioxidant ability compared with that of carnosol in oil [13,14]. Rosemary leaf extract showed better antioxidant activity than those of the other compounds, with the exception of BHA, which is a synthetic phenolic antioxidant and positive control. This is because rosemary leaf extract contains a range of antioxidant compounds, in addition to carnosol and carnosic acid.

#### 3.5.2. Oxidation Stability

**Figure 3:**
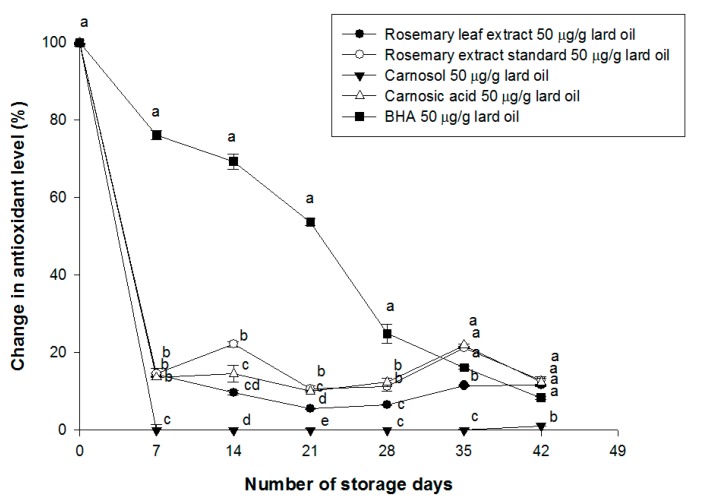
Change of residual antioxidant levels in lard oil during storage. Different letters indicate statistically significant different among the groups at *p*-value <0.05.

The residual antioxidant level in each experimental group was expressed as the percentage change of the residual level as a function of the storage period, by setting the HPLC result measured at day 0 to 100% (Figure 3). Across the storage period, the residual antioxidant level decreased significantly for all experimental groups, with the exception of BHA. 

In particular, carnosol was mostly lost during the first seven days of storage, and the residual amount of carnosol was close to 0%. After seven days, only the residual level of BHA continued to decrease, since the other antioxidants were mostly lost by the seventh day, and thereafter the residual levels remained constant. The results of the change in residual antioxidant levels in lard oil showed a similar tendency to that of the acid value and the peroxide value results. 

Thorsen & Hildebrandt (2003) reported that when carnosol and carnosic acid were dissolved in solvents, carnosol degraded more rapidly and was more unstable than carnosic acid [8]. Our results are consistent with this result. 

This experiment was performed by thermal oxidation to shorten the duration of the experiment.

As a result of the residual amount measurement, it was confirmed that the ingredients of the rosemary extract were less thermostable than the BHA, which is a synthetic antioxidant. Despite the fact that the thermal stability was low and the residual amount was decreased, it was confirmed that components of rosemary extracts showed antioxidant ability in the results of peroxide value and the acid value. In addition, considering that the food groups using rosemary extract, such as oil and sausage, are stored and distributed at room temperature or low temperature, it is considered that they will actually have an larger antioxidant effect for a longer period of time. Therefore, the rosemary extract can be used as an antioxidant.

We have determined that the oxidation rate is effectively reduced at the legal maximum of 50 μg/mL, based on the peroxide value, the acid value, and the residual antioxidant level. Since carnosic acid has a higher antioxidant capacity than carnosol, rosemary extracts with a higher carnosic acid to carnosol ratio will be a more effective antioxidant.

## 4. Conclusions

A quantitative method for the identification of rosemary extract in edible oils, processed meat products, and dressings was developed and validated using HPLC-PDA. The analytical method has been verified in accordance to the ICH guidelines. The validated HPLC-PDA analysis method is suitable for the determination of rosemary extract in edible oils, processed meat products and dressings. The validated method was verified through real sample analysis; none of the tested samples showed the presence of rosemary extracts. In addition, a functional evaluation of rosemary extract as an antioxidant was carried out. We confirmed that carnosic acid has superior antioxidative and oxidative stability compared to carnosol. These results demonstrate that the validated method is suitable for identification and quantification of rosemary extract and can be used to verify the safety of edible oils, processed meat products, and dressings that containing rosemary extracts residues. In addition, functional evaluation of the rosemary extract as an antioxidant has provided an effective use for rosemary extracts.

## Figures and Tables

**Table 1:** Correlation coefficients of the calibration curves, and limit of detection (LOD) and quantification (LOQ).

Matrix	Analytes	Range (μg/mL)	Slope	Intercept	Correlation Coefficient (R^2^) ^1^	LOD (μg/mL)	LOQ (μg/mL)
Oil	Rosmarinic acid	1.56–100	34,243.3	−27,953.9	0.9994	0.38	1.14
	Carnosol	6.25–400	7926.7	8343.5	1.0000	0.78	2.38
	Carnosic acid	6.25–400	5585.2	−13,120.1	0.9995	0.65	1.96
Processed meat products	Rosmarinic acid	1.56–100	34,954.9	−33,771.5	0.9995	0.38	1.15
	Carnosol	6.25–400	8165.6	−2906.3	1.0000	1.05	3.17
	Carnosic acid	6.25–400	5657.9	−25,961.3	0.9987	1.50	4.55
Dressing	Rosmarinic acid	1.56–100	33,781.5	−45,267.7	0.9992	0.22	0.66
	Carnosol	6.25–400	7941.4	−17,908.1	0.9997	1.39	4.21
	Carnosic acid	6.25–400	5526.4	−9656.3	0.9997	1.73	5.23

^1^ Correlation coefficient was calculated using the average of the results measured three times.

**Table 2:** Recoveries of spiked rosemary extracts (three different concentrations) from edible oil, processed meat products, and dressing (*n* = 3).

Matrix	Analytes		Concentration (μg/mL)	Mean ± SD (μg/mL)	RSD ^1^ (%)	Recovery (%)
**Oil**	Rosmarinic acid	Intra-day	5.0	4.5 ± 0.1	1.5	90.8
			10.0	7.2 ± 0.1	1.8	72.2
			20.0	14.1 ± 0.0	0.2	70.6
		Inter-day	5.0	4.9 ± 0.2	3.6	89.7
			10.0	7.1 ± 0.2	2.5	71.0
			20.0	14.3 ± 0.4	2.4	71.3
	Carnosol	Intra-day	25.0	21.4 ± 0.1	0.5	85.6
			50.0	42.4 ± 0.3	0.6	84.8
			100.0	93.5 ± 0.2	0.2	93.5
		Inter-day	25.0	20.6 ± 0.6	3.0	82.6
			50.0	4.19 ± 1.6	3.8	83.8
			100.0	93.8 ± 2.8	3.0	93.8
	Carnosic acid	Intra-day	25.0	23.1 ± 0.1	0.6	92.5
			50.0	43.6 ± 0.1	0.3	87.1
			100.0	88.6 ± 0.3	0.4	88.9
		Inter-day	25.0	22.4 ± 0.7	3.1	89.6
			50.0	42.6 ± 1.3	3.1	85.1
			100.0	88.1 ± 2.3	2.6	88.1
Processed meat products	Rosmarinic acid	Intra-day	5.0	5.4 ± 0.0	0.8	106.9
			10.0	8.7 ± 0.0	0.8	86.7
			20.0	14.7 ± 0.0	0.4	73.5
		Inter-day	3.13	5.1 ± 0.1	1.5	102.1
			12.5	8.4 ± 0.1	0.8	84.2
			25.0	14.6 ± 0.2	1.2	72.8
	Carnosol	Intra-day	25.0	27.0 ± 0.2	0.8	108.2
			50.0	53.4 ± 0.2	0.4	106.8
			100.0	99.3 ± 0.6	0.6	99.3
		Inter-day	25.0	25.4 ± 0.2	0.6	101.8
			50.0	52.2 ± 0.5	1.0	104.4
			100.0	99.1 ± 0.9	0.9	99.1
	Carnosic acid	Intra-day	25.0	28.8 ± 0.2	0.6	115.2
			50.0	55.5 ± 0.3	0.6	111.0
			100.0	101.4 ± 1.4	1.4	101.4
		Inter-day	25.0	26.8 ± 0.2	0.8	107.2
			50.0	54.5 ± 1.2	2.1	108.9
			100.0	99.4 ± 1.5	1.5	99.4
Dressing	Rosmarinic acid	Intra-day	5.0	5.8 ± 0.0	0.5	116.3
			10.0	9.5 ± 0.0	0.3	95.3
			20.0	16.1 ± 0.1	0.4	80.5
		Inter-day	5.0	5.5 ± 0.0	0.2	109.3
			10.0	9.2 ± 0.1	0.7	92.0
			20.0	16.1 ± 0.2	1.3	80.4
	Carnosol	Intra-day	25.0	28.5 ± 0.1	0.4	114.0
			50.0	52.3 ± 0.2	0.4	104.5
			100.0	101.5 ± 0.2	0.2	101.5
		Inter-day	25.0	28.4 ± 0.1	0.4	113.5
			50.0	52.1 ± 0.2	0.3	104.1
			100.0	102.8 ± 1.6	1.5	102.8
	Carnosic acid	Intra-day	25.0	29.1 ± 0.1	0.2	116.5
			50.0	51.3 ± 0.2	0.4	102.5
			100.0	99.2 ± 0.3	0.3	99.2
		Inter-day	25.0	27.7 ± 0.3	1.1	110.8
			50.0	51.0 ± 1.1	2.1	101.9
			100.0	99.3 ± 0.9	0.9	99.3

^1^ RSD is the relative standard deviation.
